# Distinct brain dynamics and networks for processing short and long auditory time intervals

**DOI:** 10.1038/s41598-023-49562-8

**Published:** 2023-12-12

**Authors:** Nicola Thibault, Philippe Albouy, Simon Grondin

**Affiliations:** 1 École de Psychologie, Université Laval, Québec, G1V 0A6 Canada; 2 CERVO Brain Research Centre, Québec, G1J 2G3 Canada; 3 International Laboratory for Brain, Music and Sound Research (BRAMS), CRBLM, Montreal, QC H2V 2J2 Canada

**Keywords:** Neuroscience, Cognitive neuroscience

## Abstract

Psychophysical studies suggest that time intervals above and below 1.2 s are processed differently in the human brain. However, the neural underpinnings of this dissociation remain unclear. Here, we investigate whether distinct or common brain networks and dynamics support the passive perception of short (below 1.2 s) and long (above 1.2 s) empty time intervals. Twenty participants underwent an EEG recording during an auditory oddball paradigm with .8- and 1.6-s standard time intervals and deviant intervals either shorter (early) or longer (delayed) than the standard interval. We computed the auditory ERPs for each condition at the sensor and source levels. We then performed whole brain cluster-based permutation statistics for the CNV, N1 and P2, components, testing deviants against standards. A CNV was found only for above 1.2 s intervals (delayed deviants), with generators in temporo-parietal, SMA, and motor regions. Deviance detection of above 1.2 s intervals occurred during the N1 period over fronto-central sensors for delayed deviants only, with generators in parietal and motor regions. Deviance detection of below 1.2 s intervals occurred during the P2 period over fronto-central sensors for delayed deviants only, with generators in primary auditory cortex, SMA, IFG, cingulate and parietal cortex. We then identified deviance related changes in directed connectivity using bivariate Granger causality to highlight the networks dynamics associated with interval processing above and below 1.2. These results suggest that distinct brain dynamics and networks support the perception of time intervals above and below 1.2 s.

## Introduction

Humans can make relatively precise and accurate judgments about the length of brief time intervals and produce speech and music with efficient timing^1^. Several theories and models have been developed to explain humans’ ability to perceive and produce time intervals^2,3^. Some theories propose that the regularity of external stimuli enables the temporal prediction of the occurrence of forthcoming events^4^. Others postulate that it is the fading of the trace of events in memory which suggests that a sense of time has elapsed^5^. Another model proposes the existence of an internal clock that keeps track of time information^6^.

In the past decades, a version of the ‘internal clock’ model, the Scalar Expectancy Theory (SET), has received growing interest in the timing and time perception literature^7^. The SET assumes that a central, unique, clock acts as a pacemaker-counter device. In theory, the pacemaker emits pulses that are accumulated in a counter, and this accumulation tracks temporal information (i.e., more accumulated pulses indicate longer perceived intervals). Moreover, the SET acknowledges that errors in time judgments do not rely solely on the properties of the pacemaker-counter device, but also on memory and decision-making processes involved in interval timing tasks^8^.

One critical feature of SET is its scalar property: the variability in a series of time judgments should increase proportionally with the magnitude of time intervals under investigation. In other words, the variability-to-time ratio should remain constant, which is not the case with time perception^9,10^. Interestingly, this ratio begins to increase when intervals are higher than 1.2 s^10–13^. For this reason, researchers distinguish the processing of sub- and supra-second intervals^14,15^. While the mechanisms for perceiving sub-second temporal intervals are thought to be associated with automatic/sensory processing^14,16^, the perception of supra-second intervals has been hypothesized to rely on executive functioning^17,18^ and attention^19,20^.

These hypotheses are primarily based on functional magnetic resonance imaging (fMRI) results. However, a persistent issue in these previous fMRI studies is the nature of the timing tasks selected for the investigations. To our knowledge, all timing fMRI studies used task-based paradigms that rely on cognitive processes such as memory^21^, decision-making^22,23^ and/or required motor responses such as button presses^24^. Consequently, these studies report inconsistent brain networks associated with sub- and supra-second processes, and, most importantly, it is not possible to determine if the differences observed depend on the temporal or the cognitive components of the tasks. Another limitation of fMRI is its limited temporal resolution compared to other imaging techniques such as EEG, and this limitation is particularly important for the study of time perception at the sub- and supra-second time scales^25^.

An even more critical problem of the current literature is the definition of the sub- vs. supra-second timing ranges. Many studies simply use the popular terminology “sub- and supra-second” to define the length of their intervals (e.g. Lewis and Miall^16^ with 0.6 s intervals for sub-second and 3 s for supra-second). Such studies could have simplified and narrowed the difference between interval length by considering robust psychophysical findings showing that any transition between “sub-” and “supra-” second timing is not exactly at 1 s^9–11,13,26^.

Notably, it has been reported that the so-called Weber fraction is not constant for time perception^9,10,27–29^. Indeed, the variability-to-time ratio begins to increase when intervals are slightly longer than circa 1.2 s^11^, or around 1.5 s^13^. This critical point was identified mainly on the basis of the non-constancy of the Weber fraction, i.e., a violation of the scalar property for time. Indeed, several studies identify a violation of this scalar property in interval timing occurring around 1.2–1.5 s (see the composite figure in Gibbon et al.^13^). In one of their studies, Grondin et al.^29^ employed a discrimination task with five distinct interval lengths (0.7, 1, 1.3, 1.6, and 1.9 s) to investigate when explicit counting becomes a viable strategy. They found that explicit counting becomes useful when intervals are longer than 1.18 s, which likely indicates a temporal limitation of the timing process used with brief intervals. Along the same line, Grondin et al.^30^ observed clear benefits of explicit counting in the 1.6 s condition but not in the 0.8 s condition. Grondin et al.^12^ have also identified that counting numbers with an 0.8 s pace (1 count every 0.8 s) lead to much less variability than counting with a 1.6 s pace. Other authors have also identified that at certain points between 1 and 2 s, Weber’s fraction is not constant as it got higher with longer intervals^27,28^. Furthermore, recent studies have also converged towards this clarification of timing for sub- and supra-second timing^31,32^.

The present study aims to determine the brain networks and dynamics associated with above and below 1.2 s interval perception. To this end, we used a passive oddball paradigm instead of an explicit timing task. In both conditions, deviant intervals could be either shorter (early) or longer (delayed) than the standard interval. The main advantage of this passive oddball paradigm is that it does not require explicit judgments about time, judgments that would involve a contribution of memory, motor, or decision-making processes.

We expected to identify distinct deviance detection-related Event related Potentials (ERPs) for empty time intervals above and below 1.2 s. We first make the hypothesis that a Contingent Negative Variation (CNV) with generators in the Supplementary Motor Area (SMA) and a cognitive network will be observed for the above 1.2-s condition but not in the below 1.2-s condition before the onset of the deviant stimuli. This hypothesis is based on Macar et al.^33,34^ who reported that the CNV is associated with decision making^35^, temporal expectation^36^, and motor preparation processes^37^, thus relying on a more cognitive network, similar to supra-second interval processes^14^. Second, based on the fMRI studies reported above, we hypothesize that i) deviance detection for intervals above 1.2 s will be observed in central executive brain regions, and that ii) deviance detection for intervals below 1.2 s will be observed in sensory networks. Finally, according to the SET, we also expect to observe higher ERPs amplitude associated with deviance detection for delayed deviants (i.e., more pulses accumulated) than for early deviants, which are intervals shorter than the standards.

## Results

Whole-brain analyses (sensor and source levels of Deviants vs. Standard contrasts) of EEG activity were performed using permutation testing and cluster randomization statistics in time and space domains (see “Methods” Section, 1000 permutations, sensors alpha = 0.05, CNV sources cluster alpha = 0.01, N1 sources cluster alpha = 0.005, P2 sources cluster alpha = 0.001, k ≥ 30 vertices for sources) as implemented in Fieldtrip (www.fieldtriptoolbox.org/). These analyses were done on the ERPs data for periods including the peaks of the N1, P2, and the CNV ERPs components (see “Methods” Section).

**Figure 1:**
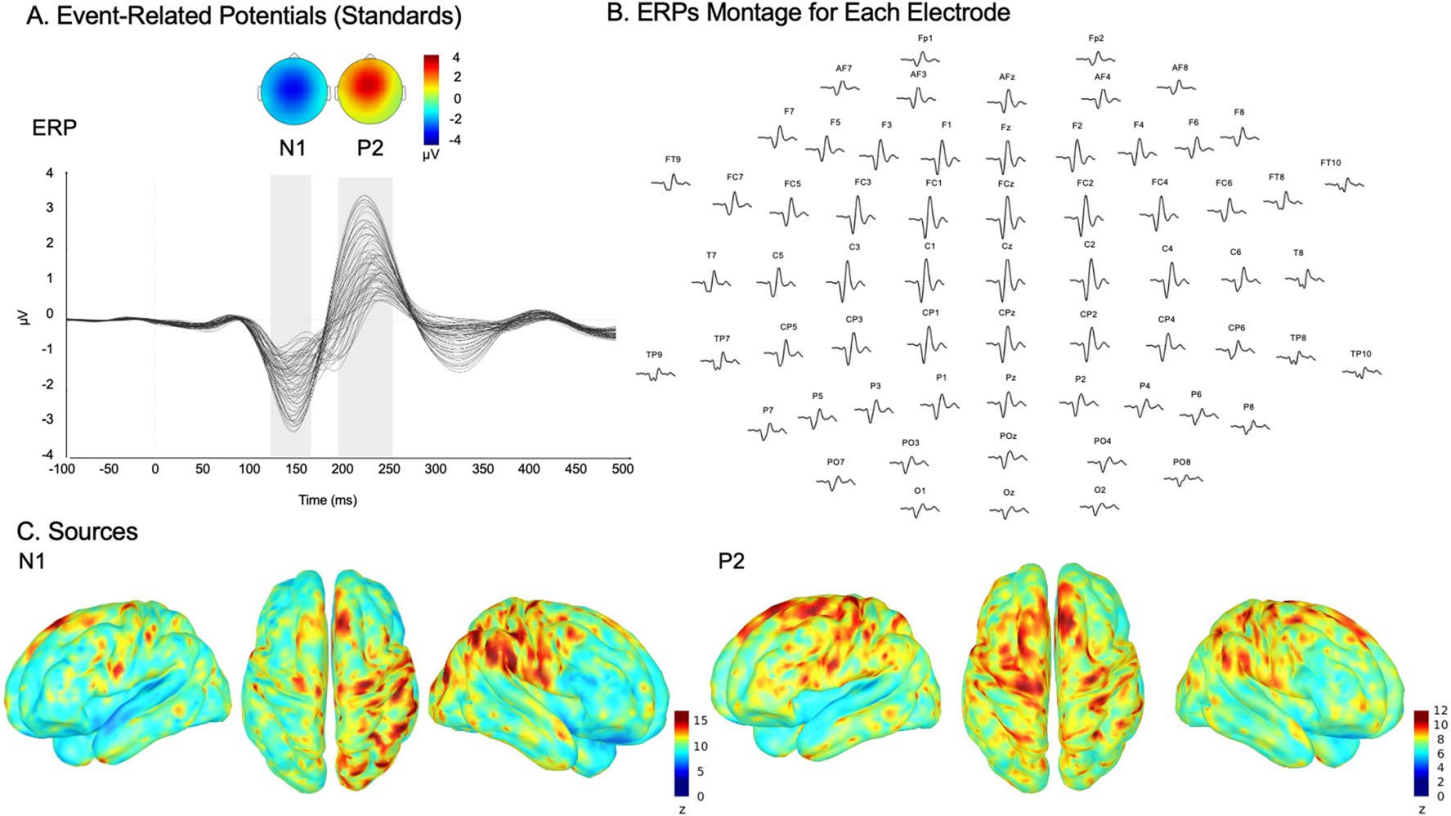
ERPs and sources for standards intervals. (**A**) Event-related potentials for standard intervals. The grand average of the signals (all electrodes) for a trial time window (− 100 to 500 ms) for the standard intervals collapsed across participants and conditions (below and above 1.2 s). Topographies represent N1 and P2 components (grey shading). (**B**) ERPs montage displaying the position of all electrodes (− 100 to 500 ms). (**C**) Cortical surface renditions show bilateral generators (z-score relative to baseline − 100 to 0 ms) for the N1 and P2 periods (grey shading in panel A).

ERPs of all standards averaged across participants showed classic frontocentral negativity for the N1 component and a frontocentral positive peak for the P2. Both N1 and P2 components were typically centered around the FCz electrode (Fig. 1A). The FCz electrode was selected for illustration. Also, as stated by Macar and Vidal^33^ most of the timing and time perception-related ERPs are generated in frontocentral areas (CNV, P300, Mismatch Negativity (MMN), and N1-P2). However, it is important to note that all analyses reported below were conducted at the whole-brain level with corrections for multiple comparisons in time (samples) and space (electrodes/vertices) domains and were thus not performed solely on electrodes of interest. Source reconstructions for the averaged time windows centered on the peak of each component (standard intervals, N1, 135–145 ms; P2, 200–210 ms) were computed (z-scored with baseline activity) to reveal expected distributed networks including auditory, inferior frontal and parietal regions for the N1 component and a parieto-frontal network for the P2 component (Fig. 1C).

**Figure 2:**
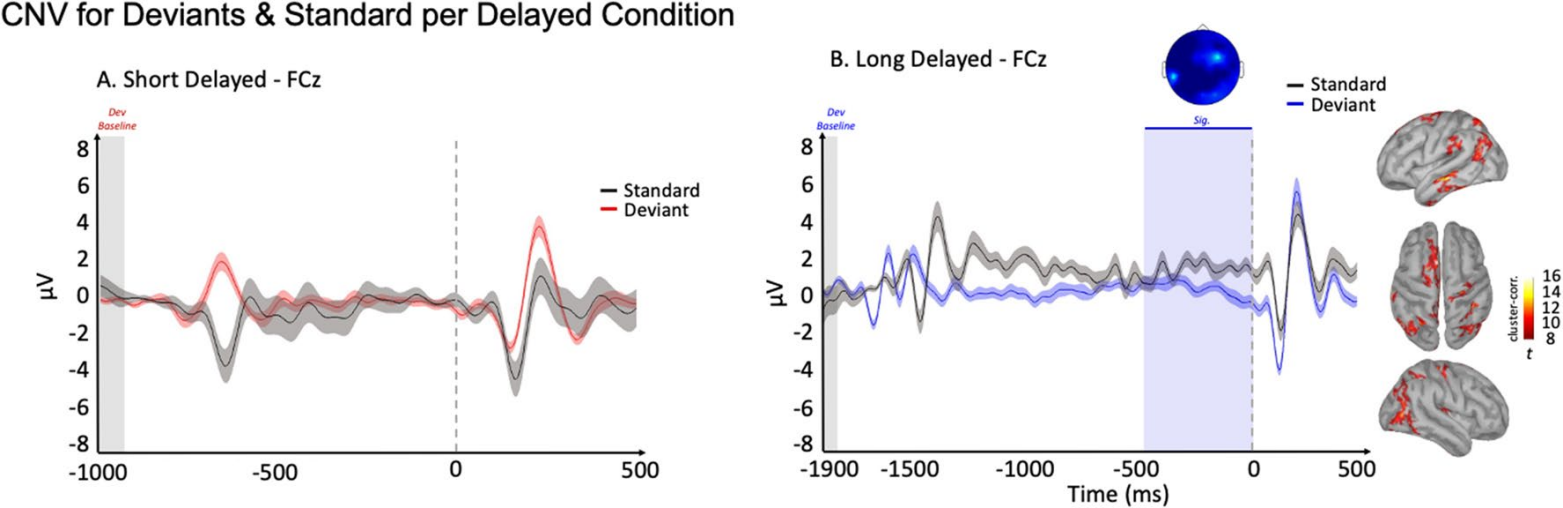
ERPs and sources for deviants and standards for the CNV. ERPs of Standards (in black) and deviants for below 1.2-s (**A** deviants are in red) and above 1.2-s (deviants are blue) conditions on the FCz electrode averaged across trials and participants. Deviants and standards are baseline corrected with a period between -100 and 0 ms relative to the onset of the previous standard sound. Shadowed lines illustrate the standard error of the mean. Raw data have been band-passed filtered between 1 and16 Hz to better isolate the CNV. Blue vertical shadowing illustrates significant period (**B**.) results at the source level (cortical meshes) are illustrated for the Deviants vs. standard contrast for the peak of CNV timing in the above 1.2-s condition (*p* < .05 cluster-corrected, alpha = .01, k ≥ 30).

We first investigated whether a CNV was elicited before deviance detection. CNV for time estimation has been associated with temporal accumulation and preparation processes^38^. Along these lines, we performed the CNV analysis for delayed deviants only, using cluster corrected (in time and space) non-parametric permutation tests with 1000 permutations and a cluster alpha of 0.05. This analysis showed a Contingent Negative Variation between − 500 and 0 ms before the onset of the delayed > 1.2-s deviant onset (*p* = 0.006, see Fig. 2B) but not for the delayed < 1.2-s deviant onset. We then performed the same contrasts at the source level (average of source activity in time for the significant time window reported in Fig. 2B) with an alpha of 0.01 (k ≥ 30). The generators of the CNV were the left SMA, left secondary AC, left inferior temporal cortex, right AC, and bilateral parietal cortices (Fig. 2B).

**Figure 3:**
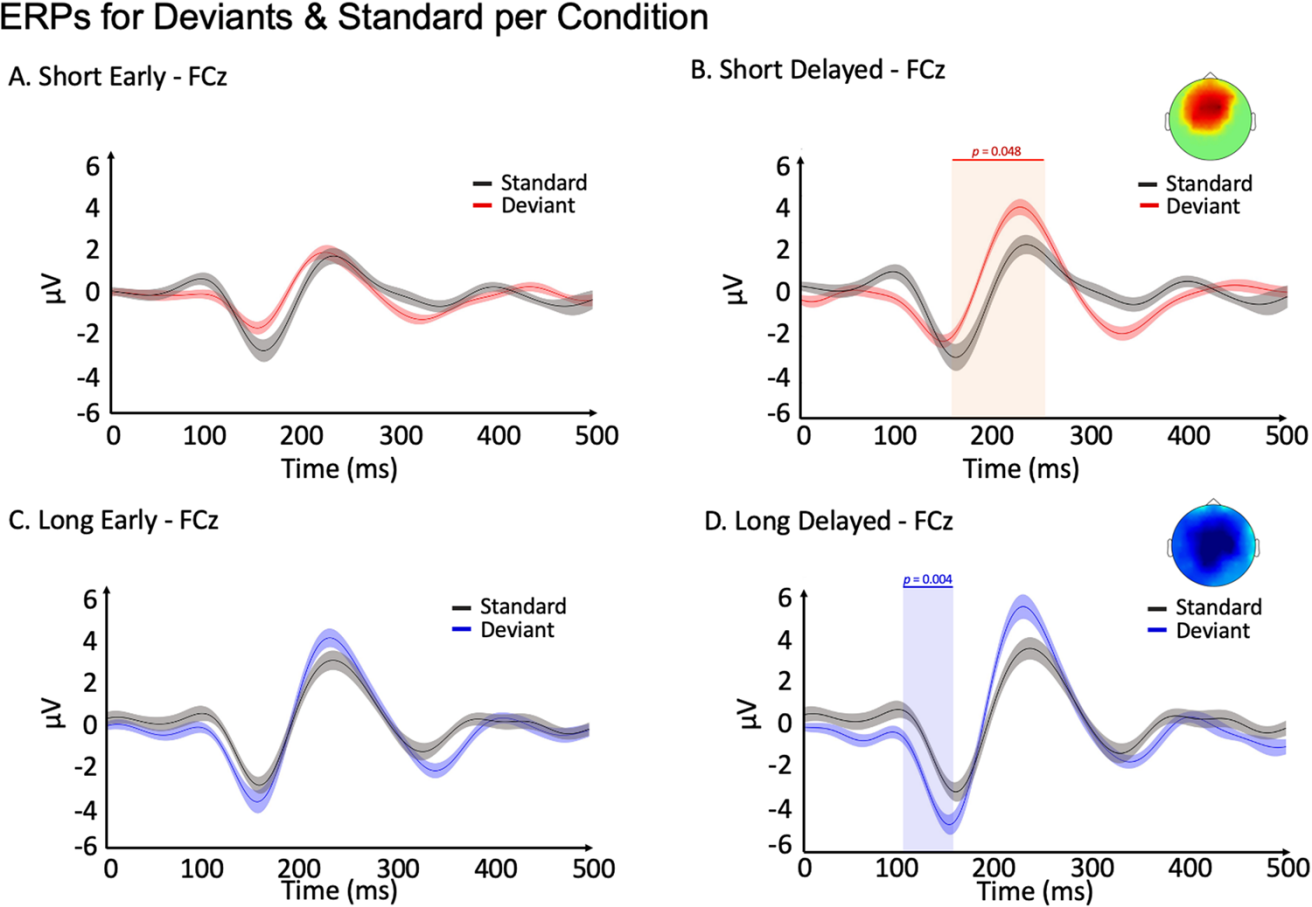
Deviants versus Standards at the sensor level: ERPs of Standards (in black) and deviants for below 1.2 s (**A**. early, **B**. delayed, deviants are in red) and above 1.2 s (**C**. early, **D**. delayed, deviants are in blue) conditions on the FCz electrode averaged across trials and participants. Shadowed lines illustrate the standard error of the mean. Cluster-corrected topographies of significant differences are displayed for the significant effects (< 1.2-s delayed and > 1.2-s delayed conditions). Blue and red vertical shadowing illustrate significant periods.

We then investigated the brain responses associated with deviance detection for above and below 1.2-s time intervals after the onset of the deviant sound. Non-parametric permutation tests with cluster-based correction in time and space were performed for below and above 1.2-s deviant ERPs against their respective standard ERPs separately for a time window of − 100 to 500 ms post-stimulus onset (see Fig. 3). Significant differences between deviants and standards were observed for the < 1.2-s delayed condition during the P2 time window (*p* = 0.048, for 164 to 254 ms, Fig. 3B) and during the N1 period, suggesting a difference in the N1 for the > 1.2-s delayed condition (*p* = 0.004, for 106–168 ms, Fig. 3D). This difference was observed few milliseconds before the N1 peak, thus corresponding to the N1 time-window^39^. Finally, note that no increased negativity was observed after the N1 peak when contrasting deviants to standards for all conditions, suggesting that no MMN was elicited by the deviance in the present study^39,40^.The P2 component observed for the > 1.2-s delayed condition was not significant (*p* = 0.089). Cluster-corrected topographies were calculated for the above and below 1.2-s delayed deviants against their respective standards. When considering only early deviants, ERPs amplitudes were not significantly different from the standards (Fig. 3A,C).

**Figure 4:**
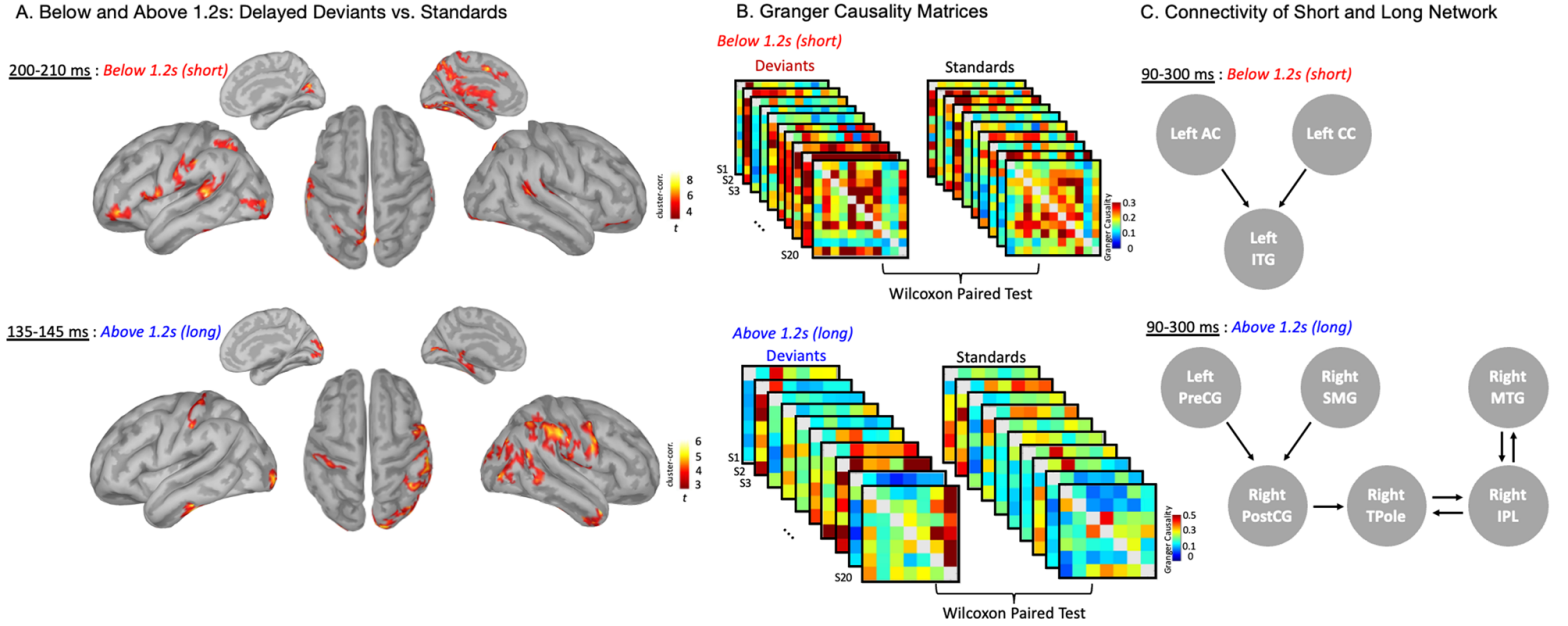
Results at the source level for Below and Above 1.2-s Deviants vs. Standards ERPs and Granger causality results. (**A**) Deviants (delayed) vs. standard contrast at the source level (*p* < .05 cluster-corrected, alpha (> 1.2 s) = 0.005, alpha (< 1.2 s) = 0.001 k ≥ 30) for the below 1.2-s (short) condition (P2 time window, top panel) and above 1.2-s (long) condition (N1 time window, lower panel). (**B**) N × N Granger causality connectivity matrices for each subject (Maximum GC, model order = 2, 90–300 ms, 11 × 11 for < 1.2 s and 7 × 7 for > 1.2 s) for deviants and standards. Wilcoxon Paired Test was performed between connectivity matrices of deviants and standards for each condition. (**C**) Arrows indicate significant differences between deviants and standards for the below 1.2-s condition (delayed, upper panel) and the above 1.2-s condition (delayed, lower panel). AC: Auditory Cortex; CC: Cingulate Cortex; ITG: Inferior Temporal Gyrus; PreCG: Precentral gyrus; PostCG: Postcentral Gyrus; TPole: Temporal Pole; IPL: Inferior Parietal Lobule; MTG: Middle Temporal Gyrus.

Additionally, the same analyses were conducted at the source level with cluster-based non-parametric permutation tests with an alpha of 0.005 (for > 1.2 s condition) and alpha of 0.001 (for the < 1.2-s condition) and 1000 permutations with k ≥ 30 (N1 for > 1.2 s: average in time between 135 and 145 ms; P2 for < 1.2 s: average in time between 200–210 ms). When deviance occurred in the > 1.2-s condition, deviance detection-related activity was observed for the left superior parietal lobule, left inferior postcentral gyrus, left parsopercularis (posterior inferior frontal gyrus: IFG), bilateral parsorbitalis (anterior IFG), left auditory cortex, left inferior temporal gyrus (ITG), left cingulate cortex (CC), left SMA, right middle temporal gyrus (MTG), and right auditory cortex (*p* < 0.05). When deviance occurred in the < 1.2-s range, deviance detection-related activity arose in the right precentral gyrus, left postcentral gyrus, left ITG, left parahippocampal gyrus, right inferior parietal lobule (IPL), right MTG, right supramarginal gyrus (SMG), and right temporal pole (TP) (*p* < 0.05, see Fig. 4A).

Finally, we investigated the changes in directed connectivity between the regions that participated in deviance detection in each condition. Granger causality NxN analysis (Fig. 4B,C) was conducted using the algorithms (see “Methods” Section) implemented in Brainstorm^41^. Using the regions that were significant in Fig. 4A, paired Wilcoxon tests were performed to contrast the Granger causality connectivity matrices of deviants and standards for each condition (Fig. 4B). Results show that for the below 1.2-s condition, deviance detection is associated with increasing communication from the left auditory cortex and left cingulate cortex to the left inferior temporal gyrus (see Fig. 4C). For the above 1.2-s condition, deviance detection is associated with increasing communication from the left postcentral gyrus and the right SMG to the right postcentral gyrus. The right precentral gyrus then connects to the right temporal pole which sends and receives information from the right IPL. Finally, the right IPL sends and receives information from the right MTG (see Fig. 4C).

## Discussion

### Identification of auditory event-related potentials (aERP)

Standard ERPs were thoroughly identified to ensure good data quality (Fig. 1). We observed classic fronto-central N1 and P2 components in response to the sounds, with activity centered around Fz, Cz, and FCz electrodes^42–46^. Source reconstruction of the N1 component revealed generators in the right auditory cortex, and the right postcentral and precentral gyri/IFG. These generators of the N1 component are in line with previous studies and are typical of the aERP^47–49^. The P2 component is generally more distributed than the N1 component with generators in the bilateral frontal lobes, the bilateral parietal lobes, and the bilateral auditory cortices, which is also in accordance with the literature^46^.

### CNV as a marker of temporal expectancy for > 1.2-s delayed intervals

We first identified a CNV only in > 1.2-s conditions (delayed, see Fig. 2) with generators in SMA and parietal regions. Macar et al.^34^ have also identified a CNV in above 1.2 s time intervals (1.8–3.2 s). They suggested that the SMA may act as the temporal accumulator, essentially operating as working memory for temporal information. This would be in line with the idea that cognitive mechanisms are involved in the processing of long temporal intervals^14^. Interestingly, the CNV was not observed for the < 1.2 s condition (Fig. 2A). This is in line with many studies such as the one of Liu et al.^50^ supporting the idea of the CNV being elicited solely for long intervals. This is because the CNV’s amplitude typically increases as a function of the attention paid to the task. Cognitive functions, such as attention, have been thoroughly associated with supra-second timing. In brief, our results are consistent with the ideas of Liu et al.^50^ and Lewis and Miall^14^ regarding the role of cognitive mechanisms when processing long (say, supra-second) intervals.

Activity in the auditory regions has also been previously linked to the CNV related to temporal expectancy in auditory tasks^51^. Moreover, our results suggest the implication of a parietal-frontal network (in delayed condition) for the generation of the temporal CNV. Parieto-frontal network involvement during the CNV has previously been identified to play a key role in endogenous attention^52^, which would also be in line with the idea that the processing of long time intervals requires cognitive control. Finally, we identified the involvement of the temporal cortex which acts as the hub of memory and working memory^53–55^. This is in line with previous studies suggesting that the CNV can be considered as a marker of temporal accumulation. In the present study, results suggest that the CNV generated by the SMA coupled with the temporal cortex acts as working memory for temporal information, holding temporal details into short term memory for further processes such as decision-making^33^.

### Distinct waveforms of deviance detection for below and above 1.2-s processing

When comparing the ERPs of above and below 1.2-s deviants to their respective standards after the onset of the deviant sound/interval, two distinct significant waveforms were observed. The first emerged early after the deviance (negativity around 100 ms) and was significant only for the delayed > 1.2-s condition (see Fig. 3D). The second component emerged later (positivity around 200 ms) and was only significant for the delayed < 1.2-s condition (see Fig. 3B). Even if the P2 component was only significant for the below 1.2-s condition, it is relevant to point out that a similar trend, though non-significant, was observed for the above 1.2-s condition (see Fig. 3D). This (almost) P2 for the above 1.2-s condition seems to have similar generators to the one of the below 1.2-s condition. Indeed, between-condition contrasts are significant only for N1, not for P2 (as shown in Supplementary Fig. 1).

These two distinct components related to deviance detection are coherent with components identified during human error monitoring^56^. The processing of error-related information can be dissected into two different components: error-related negativity (ERN/Ne) and error-positivity (Pe). The former is a fronto-central negative component peaking around 20–130 ms after an error detection, and the latter is a positive fronto-central component peaking around 200–500 ms^57–59^. The ERN/Ne appears during error detection^60^, correction, and compensation^61,62^. The latter, the Pe component, is elicited for conscious and easier error processing^63,64^. Observing a Pe component only for the < 1.2-s condition in the present study is in line with previous work suggesting that shorter intervals may be easier to estimate accurately than longer intervals, as the Weber fraction in timing perception is increasing when intervals are longer than circa 1.2–1.5 s^11,13^.

Furthermore, our results show that for the < 1.2-s condition, the latency of the significant component (P2) arrives later than for the > 1.2-s condition (N1). We propose that the reason for this lies in the role of these two components for timing and time perception. Indeed, the N1 component has been linked to attention mechanisms in the expectation of temporal events^65^. Indeed, the level of attention oriented towards a target modulates the N1 amplitude^66,67^. The higher the attention level, the larger the amplitude of the N1 component, which would be in line with the idea that the processing of intervals above 1.2 s requires more cognitive control.

On the other hand, increases in P2 have also been identified with sensory gating, a process which filters irrelevant information from reaching higher-order cognitive functions^68,69^. In the present case, P2 is elicited during deviance of below 1.2-s time intervals, which indicates it may have passed sensory gating and made it to consciousness^68,70^. Our results are in line with the idea that below 1.2-s timing is automatic and sensory oriented^16^.

### Delayed deviance allows proof of error accumulation

As shown in Fig. 3, only the deviances occurring after the expected standard (delayed deviants) were significantly detected. No effect was found for the early deviants. This finding is in line with the pacemaker-counter model of time perception^8^, where an accumulation of pulses tracks the time that has passed. This suggests that in the delayed deviant condition, participants accumulate more pulses in the counter than they usually accumulate with a standard presentation, which translates into further evidence that an error has occurred. In contrast, in the early deviant condition, the accumulation of pulses does not exceed the standard’s pulse count, which may not allow for error detection. This may explain why only the ERP amplitude was significant, in both the below and above 1.2-s conditions when longer rather than shorter deviants were presented.

### Lack of mismatch negativity

A surprising result in the current study is the absence of MMN. Indeed, no increased negativity (after the N1 peak) was observed when contrasting deviants against standards for all conditions. Typically, the MMN is generated when there is a violation of an expected event in an expected sequence^71^. In the present study, no MMN was elicited when a deviant empty time interval was presented. One possible interpretation is that the stimuli were processed as a train of empty time intervals which created shifts in the timing of the N1 (for > 1.2 s) and P2 (for < 1.2 s) components. The delayed sound may have been interpreted as an omission, and not as a violation of expectation, which would explain why there was no MMN^72^.

### Networks underlying below and above 1.2-s processes

In the literature, there seem to be recurrent structures that are typical of time perception and independent of interval length. In our study, we show that two structures seem to be independent of timing length as they appear in both above and below 1.2-s conditions. These structures are the ITG and the postcentral gyrus.

The postcentral gyrus has been thoroughly identified in several beat perception tasks, especially when beat changes were detected^73,74^. Participants in our experiment may perceive the different trials as one long-lasting beat with some changes in the tempo (deviants) every now and then. Its activation in both conditions may suggest that the postcentral gyrus is not sensitive to length duration for implicit timing and time perception. Additionally, activity in the ITG may reflect a violation of the temporal expectation. Indeed, the ITG has been linked to sequence or expectation violations in various domains such as learned visual sequence and semantic violations^75,76^.

### Networks underlying above 1.2-s processes

As shown in Fig. 4, between 135 and 145 ms after stimulus onset, the ERN/Ne component observed in the delayed > 1.2-s condition (but not the early condition) originates from the left postcentral gyrus, right SMG, right postcentral gyrus, right temporal pole, right IPL, and right MTG. The SMG activation is coherent with the processing of longer intervals being associated with motor processes. Indeed, the right SMG has been consistently associated with motor preparation processes^77,78^, consolidating the idea that the processing of supra-second intervals is closely linked to motor processes. The motor cortex (precentral gyrus) is known to play a role in beat perception and time perception^3,79,80^. The motor cortex coupled with a parietal activation (in the present case, the IPL) has been identified as a network used in the mental simulation of action^81–83^. Our results are in line with the idea that the processing above 1.2-s intervals may require motor processes to maintain a rhythm with longer intervals. It can be argued that, in previous studies, the motor cortex may have been falsely identified in below 1.2-s timing ranges because of the necessity to use a motor response in most of the fMRI timing tasks. Regarding the activation in the temporal pole, it has been associated with higher cognitive functions such as autobiographical memory^84^ and auditory memory^85^. This highlights the role of cognitive processes, such as memory, in keeping track of certain auditory stimulus characteristics (such as length) in longer-timing tasks.

Lewis and Miall^16^ suggested that the left IPL plays a key role in time perception in the longer (above 1.2 s) duration ranges. The inferior parietal lobes are known to play a role in the orientation of attention and working memory^86–88^. Thus, it is not surprising to observe that these structures are involved in the processing of > 1.2-s intervals, *a fortiori* considering that processing such intervals is known to be cognitively oriented. Regarding the activity observed in the parietal cortex, similar findings have been reported by Hayashi et al.^89^ who showed that the parietal inferior lobule is associated with duration-tuning. When a specific duration is repeated, the activity of the IPL is reduced. However, it is higher when the difference between durations is increased and thus might explain its activation in the processing of > 1.2-s intervals only.

The data also indicate activation in the right MTG during the processing of intervals longer than 1.2 s. Lesions in the medial temporal lobe are known to lead to deficits in time orientation, suggesting the importance of this region in timing and time perception^90^. Temporal orientation is a cognitive process which allows for one’s sense of time. The MTG has also been thoroughly identified in memory functions, such as working memory^91^, which would be consistent with the idea that longer interval processes are cognitively driven.

Finally, connectivity results demonstrate that, for the above 1.2-s condition, increased communication during deviant perception relative to standard was observed from the left precentral gyrus and the right SMG to the right postcentral gyrus (Fig. 4C). This is in line with the fact that the timing of longer intervals requires further motor preparation. Indeed, these two structures are involved in motor process and preparation^3,77^. The right postcentral gyrus then further integrates this motor timing information as rhythmic stimuli^73^. Deviance detection is then sent to the temporal pole as it processes the rhythmic sequence into very short-term auditory memory^85^. Then, the right IPL processes the temporal information and plays a key role in interval tuning^92^. The right IPL processes this auditory sequence and flags whether the length of the interval is different from what was stored in the temporal pole. Finally, the right MTG further integrates this information and stores it in working memory^91^.

These findings for the supra-second range of timing are in line with the cognitive model of timing in the > 1.2-s duration ranges^19,20^ which underlies a contribution of the central executive network.

### Networks underlying below 1.2-s processes

As shown in Fig. 4A, at around 200–210 ms, the positive component observed only in the delayed < 1.2-s condition was localized in the left auditory cortex, the SMA, the cingulate cortex, the IFG, the ITG, and the superior parietal lobule. The critical role of the SMA in time perception is well known^23,93–95^. In the present study, the SMA operates only in the < 1.2-s timing condition, during the P2. Coull et al.^96^ have identified the SMA’s implication in perceptual timing and error monitoring, which is in line with the demands of our experiment. Indeed, Spieser et al.^97^ applied anodal transcranial Direct Current Stimulation (tDCS) to the preSMA, during a conflict task in which participants were impulsively selecting an incorrect response. Stimulation of the (pre)SMA reduced the number of overt errors. This suggests that the (pre)SMA plays an active role in selectively suppressing unwanted responses or stimulations. This suggests that the SMA may play a role of selectively identifying deviance only in the < 1.2-s range. The coactivation of the IFG and the auditory cortex suggests that the processing of < 1.2-s intervals is very close to auditory sensory processes. Indeed, the IFG and auditory cortex have previously been identified to play a role in auditory processes of integration, especially in music^48,98^, language and speech perception^99–101^. Regarding the left parietal cortex, Wiener et al.^102^’s meta-analysis identified this region as essential to implicit timing and part of some sensorimotor processing. Finally, the cingulate cortex’s activation has also been previously identified in sub-second timing^103^. In the present study, we propose that the cingulate cortex’s activation may have a role in the monitoring aspect of deviance, especially in the < 1.2-s interval condition where a more sensory network is involved^104^. Indeed, its activation may be related to conflict monitoring, signaling to the brain the need for greater cognitive control after deviance detection.

Connectivity results demonstrate that, for below 1.2-s, both the left auditory cortex and the left cingulate cortex convey information to the left ITG (Fig. 4C). We propose that deviance detection is first processed by the auditory cortex and that the temporal aspect of deviance is first integrated by the cingulate cortex^104,105^. Then, the ITG integrates this violation of a learned sequence^76^. These results are in line with a sensory-oriented process of sub-second timing, but it does not seem to validate the commonly reported motor-oriented processes of sub-second timing^16^.

### Conclusion

This study provides direct evidence for the existence of distinct time perception mechanisms for processing intervals below and above 1.2 s. An important contribution of the present study is the difference in the processing latency of < 1.2-s and > 1.2 s-time intervals. Intervals longer than 1.2 s are processed rapidly (135–150 ms post-stimulus) and intervals shorter than 1.2 s are processed later (200–245 ms post-stimulus). Mostly consistent with the literature, a passive time perception paradigm involving short intervals exclusively recruits a sensory network. This result is in line with the idea that processes behind the perception of short intervals are mostly automatic^16^. On the other hand, intervals longer than 1.2 s recruit a more active and cognitive network. This result is also coherent with the literature describing the need for cognitive support during the processing of longer intervals^14,16^.

Furthermore, in both duration conditions, deviants occurring after the expected standard always elicit a higher amplitude cortical response, translating in more accumulated evidence of deviance occurrence, which is in line with the pacemaker-counter model of time perception^8^. This suggests there is a common pacemaker-counter mechanism for processing intervals shorter and longer than 1.2 s. However, differences in the mechanisms involved in the processing of intervals above and below 1.2 s lie in the cortical responses’ components, the latency of the process, and their localizations.

## Methods

### Ethics

All procedures were approved by the Ethics Committee of the CIUSSS de la Capitale Nationale: 2021-2156. All procedures were carried out in accordance with relevant guidelines and regulations of the Ethics Committee of the CIUSSS de la Capitale Nationale: 2021-2156.

### Participants

Twenty healthy participants (10 females and 10 males; mean age, 23.15; SD, 1.50; age range; 21–27, mean education, 17.63 years; SD, 2.16; education range, 14–22 years; 17 right-handed and 3 left-handed), all with normal hearing, no history of neurological or psychiatric disorders, gave their informed consent to participate in this study and received monetary compensation for their participation.

### Task and procedure

Participants were scheduled for two sessions lasting approximately an hour and a half each (52 min of experiment and approximately 30 min of EEG preparation and set-up). Over the two sessions, participants went through twelve 8-min blocks of an oddball paradigm with time intervals.

Each 52-min session consisted of 6 blocks. Each block was separated by a 3-min break. The task consisted in a passive listening of an auditory oddball paradigm; eyes opened with a fixation cross displayed on the screen. For each block, 40 trials of 10 sounds were presented for each condition (below and above 1.2 s). It is relevant to note that all trials were presented continuously without inter-trial intervals, which results in a continuous flow of stimuli (corresponding to a classic oddball paradigm). For each trial, a deviant empty time interval appearing between sounds was pseudo-randomly presented across the standard empty time intervals. The standards and deviants are referring to empty time intervals presented. In a series of 10 empty time intervals, 9 empty time intervals were “standards” (which corresponded to 0.8 s in the < 1.2-s condition, and 1.6 s in the > 1.2-s condition) and one was “deviant” (the empty time interval was either shorter/early) or longer/delayed). There was a repetition of at least 4 standard time intervals before a deviant time interval was presented. For half of the blocks, the < 1.2-s condition trials were presented first, and for the other half, the > 1.2-s condition trials were presented first. The deviant empty time interval for each sequence of ten empty time intervals was selected at random. The deviant empty time intervals were either early (< 1.2 s: 0.70, 0.75 s; > 1.2 s: 1.4, 1.5 s) or delayed (< 1.2 s: 0.85, 0.9 s; > 1.2 s : 1.7, 1.8 s) as compared to the empty standard interval. Empty time intervals, by opposition to filled intervals, were used to avoid auditory fatigue. Furthermore, for the range of durations used in the current experiment, empty intervals are at least as easily discriminated as filled intervals^106,107^. This resulted in 60 deviants for each condition. Note that 60 standard trials were randomly selected to perform contrasts between deviant and standard (see below). The participants were instructed to listen to the different sounds that would be presented.

All sounds were 5 kHz and lasted 30 ms, with a 10-ms ascending-descending envelope. Presentation software (Neurobehavioral Systems, Albany, CA, USA) was used for the delivery of the experimental protocol and to trigger auditory stimuli. The sounds were presented with Audiotechnica ATH-M50x at 70 dB (SPL). For the six blocks of one given session, the < 1.2-s condition and the > 1.2-s condition were presented in alternation; and then the opposite sequence was adopted for the six blocks of the other session. Auditory event-related potentials (ERP) for each condition were then studied at the sensors and source levels.

### EEG recording

A 64-channel EEG cap with active electrodes (ActiCap—Brain Vision Solutions, Montreal) was used to capture the electroencephalographic activity with two 32-BrainAmp MR Plus amplifiers (Brain Products, Munich, Germany). The installation of the EEG was completed with respect to the standard 10–20 installation. The signal was band-pass filtered between DC and 1000 Hz and digitized at a sampling rate of 1000 Hz.

All channels were referenced with an electrode placed on the nose and with a forehead ground. All electrodes had an impedance of < 20 kΩ. EEG data were preprocessed using Brainstorm software^41^ combined with Fieldtrip functions (http://www.fieldtriptoolbox.org/) and MATLAB (MathWorks, https://www.mathworks.com/products/matlab.html). The EEG preprocessing included notch filtering of the wall outlets’ contamination (removed the 60, 120, and 180 Hz). A band-pass filter for frequencies of interest (ERPs) between 2 and 16 Hz was applied for the N1-P2 preprocessing and between 1 and 16 Hz for the CNV preprocessing. The filtered data were subjected to Independent Component Analysis (ICA) using EEGlab functions (https://sccn.ucsd.edu/eeglab/). ICA removes muscle artefacts such as blinking and eye movements. Using time-course and topographic information, components representing clear ocular artefacts were identified and removed from the filtered data. Each event (deviant and standard) was inspected from -1900 ms to 1900 ms relative to the onset of each sound, and trials for which the signal varied by more or less than 150 μV during the duration of the trial were excluded: between 23 and 57 trials per condition were kept for each participant. For each epoch, a baseline correction of 100 ms (− 100 to 0) before stimulus onset was performed. Analyses for the CNV include a baseline corrected between − 100 and 0 ms relative to the onset of the previous standard ERP.

### Source modeling

Source reconstruction of the event-related potentials was performed using functions available in Brainstorm, all with default parameter settings^41^ as in Albouy et al.^108^. Forward modeling was performed using a realistic head model: Symmetric Boundary Element Method from the open-source software OpenMEEG. The lead-fields were computed from elementary current dipoles distributed perpendicularly to the cortical surface. EEG source imaging was performed by linearly applying Brainstorm’s weighted-minimum norm operator onto the preprocessed data. The data were previously projected away from the spatial components of artefact contaminants. For consistency between the projected data and the model of their generation by cortical sources, the forward operator was projected away from the same contaminants using the same projector as for the EEG data. The EEG data were projected on a cortical surface template available in Brainstorm (adult cortical surface of 15,002 vertices serving as image support for EEG source imaging). Baseline normalization (− 100 to 0 ms relative to stimulus onset) was calculated using a Z-score, and 3-mm Gaussian spatial smoothing was applied to the cortical mesh. Analyses for the CNV include a baseline for the previous standard (i.e., − 1.7 to − 1.6 s with the 1.6-s standard). ERPs were calculated separately for the standard and deviant conditions for each participant. Source reconstruction was performed for each participant for the entire ERPs time period. The ERPs at the sensor and source levels for each standard (< 1.2 s: 0.8 s; > 1.2 s: 1.6 s) and deviant type (< 1.2 s early deviants: 0.70, 0.75 s; < 1.2 s delayed deviants: 0.85, 0.9 s; > 1.2 s early deviants: 1.4, 1.5 s; > 1.2 s delayed deviants: 1.7, 1.8 s) of all participants were averaged. Only the source contrasts for the > 1.2-s condition were calculated for the CNV analyses because only the > 1.2-s condition elicited a CNV (see Fig. 2).

### Granger causality

The bivariate Granger causality values were calculated using algorithms implemented in Brainstorm^41^. Granger causality is a measure of directed functional connectivity^109,110^. The NxN Granger causality values were calculated with a model order of 2, using the maximum value of connectivity applied after the computation of connectivity measure. Bivariate Granger causality values were calculated for standards and deviants for the > 1.2-s and < 1.2-s conditions for 90–300 ms time windows for the regions of interest identified in Fig. 4A. For the < 1.2-s condition, bivariate Granger causality values were calculated for left superior parietal gyrus, left inferior postcentral gyrus, left parsopercularis (posterior IFG), left parsorbitalis (anterior IFG), left auditory cortex, left inferior temporal gyrus, left cingulate cortex, left supplementary motor area, right middle temporal gyrus, right auditory cortex, and right pars orbitalis (11 × 11). For the > 1.2-s, values were calculated for the right precentral gyrus, left postcentral gyrus, left inferior temporal gyrus, left parahippocampal gyrus, right inferior parietal lobule, right middle temporal gyrus, right supramarginal gyrus, and right temporal pole (7 × 7). This resulted in a connectivity matrix for each subject by condition for deviants and standards.

### Statistics

Whole-brain analyses (sensors and sources of deviants vs. Standard contrasts) of EEG activity were performed using non-parametric permutation testing and cluster randomization statistics in time and space (1000 permutations, CNV sources cluster alpha = 0.01, N1 sources cluster alpha = 0.005, P2 sources cluster alpha = 0.001, k ≥ 30 for sources) as implemented in Fieldtrip (www.fieldtriptoolbox.org/). These analyses were done on the ERPs data averaged in time for two predefined time periods corresponding to the N1 and P2 ERPs components (see Fig. 4A). The same analyses (with cluster alpha = 0.05 for sensors and alpha = 0.01 for sources) were performed for the CNV time period (− 295 to − 165 ms for delayed). Moreover, paired non-parametric Wilcoxon tests were performed to contrast deviants against standards connectivity matrices per condition (> 1.2-s early and delayed) on the bivariate Granger causality values. The results of this test give information on which regions are significatively interconnected and the direction of this connection while deviance occurs for each condition.

### Supplementary Information

Supplementary Figure 1.

Supplementary Legends.

## Data Availability

Data and pipelines for analyses will be available via Université Laval’s repository link upon acceptance. https://doi.org/10.5683/SP3/IRGLHN.
