# Supplementary figures and images for: Distinct brain dynamics and networks for processing short and long auditory time intervals
Source: Sci Rep. 2023 Dec 12;13:22018. doi: 10.1038/s41598-023-49562-8 (PMC10716402; doi:10.1038/s41598-023-49562-8)

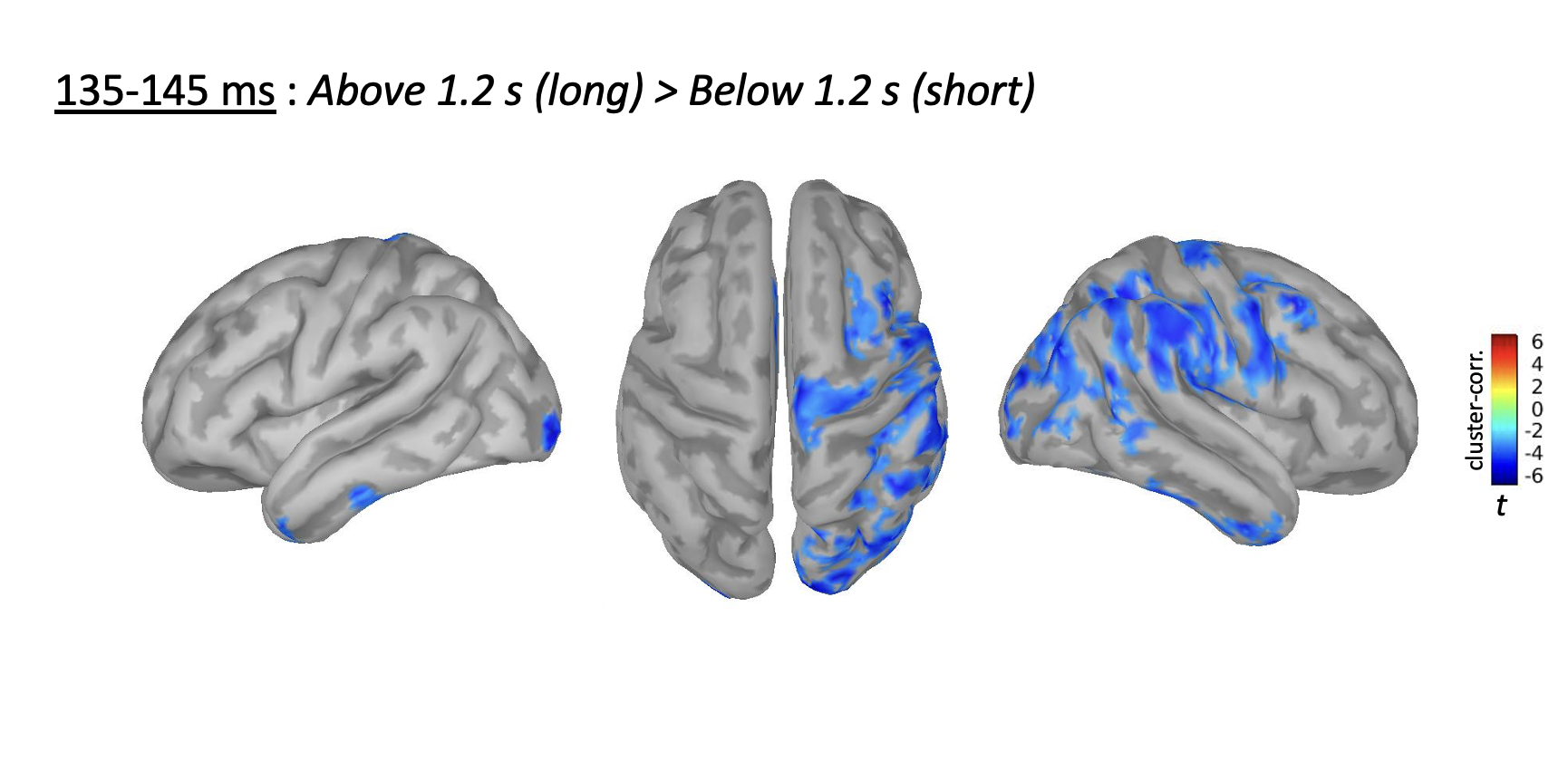

Supplement: Supplementary file 1 — Supplementary Figure 1. [file 41598_2023_49562_MOESM1_ESM.png]
