# Supplementary material for: Distinct brain dynamics and networks for processing short and long auditory time intervals
Source: Sci Rep. 2023 Dec 12;13:22018. doi: 10.1038/s41598-023-49562-8 (PMC10716402; doi:10.1038/s41598-023-49562-8)
Supplement: Supplementary file 2 — Supplementary Legends. [file 41598_2023_49562_MOESM2_ESM.docx]

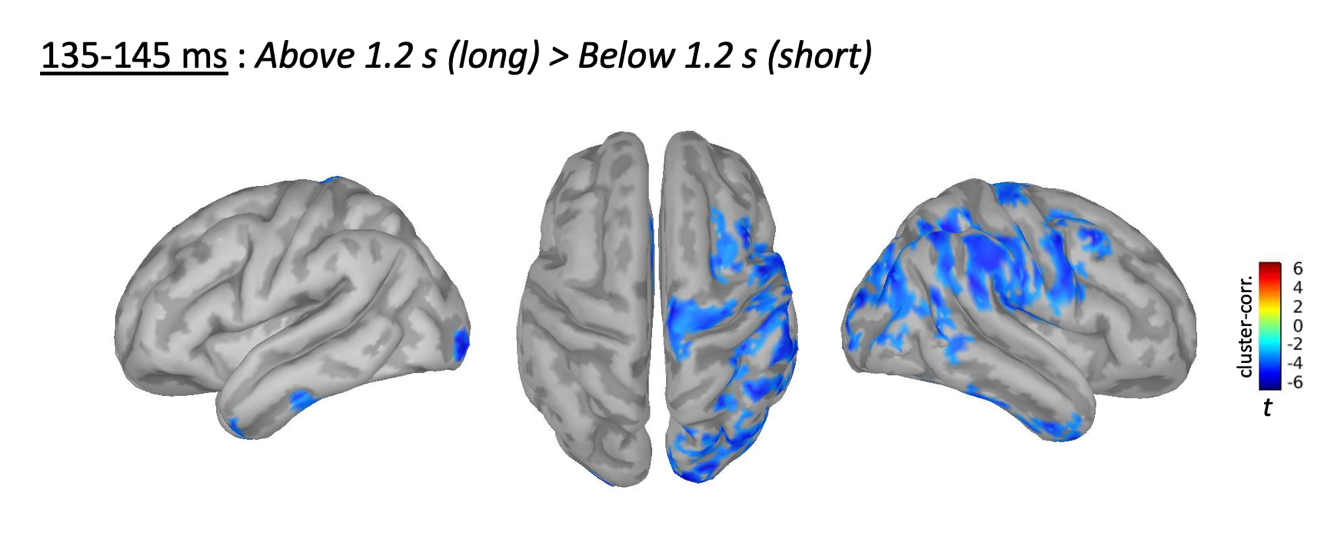


**Supplementary Figure 1. Between-conditions contrast performed at the source level between below 1.2 s and above 1.2 s difference waves (delayed).** *p* < .05 cluster-corrected, alpha = .005, k ≥ 30).
